# Carbon sequestration and soil restoration potential of grazing lands under exclosure management in a semi‐arid environment of northern Ethiopia

**DOI:** 10.1002/ece3.5223

**Published:** 2019-05-14

**Authors:** Tsegay Gebregergs, Zewdu K. Tessema, Negasi Solomon, Emiru Birhane

**Affiliations:** ^1^ Shire‐Maytsebri Agricultural Research Center Tigray Agricultural Research Institute Shire Ethiopia; ^2^ Rangeland Ecology and Biodiversity Program, School of Animal and Range Sciences Haramaya University Dire Dawa Ethiopia; ^3^ Department of Land Resources Management and Environmental Protection Mekelle University Mekelle Ethiopia; ^4^ Faculty of Environmental Sciences and Natural Resource Management Norwegian University of Life Sciences Ås Norway

**Keywords:** carbon stock, Ethiopia, exclosure, grazing land, semi‐arid, soil property

## Abstract

Exclosures are used to regenerate native vegetation as a way to reduce soil erosion, increase rain water infiltration and provide fodder and woody biomass in degraded grazing lands. Therefore, this study assessed the impact of grazing exclosure on carbon sequestration and soil nutrients under 5 and 10 years of grazing exclosures and freely grazed areas in Tigray, northern Ethiopia. Carbon stocks and soil nutrients increased with increasing grazing exclusion. However, open grazing lands and 5 years of grazing exclosure did not differ in above‐ and belowground carbon stocks. Moreover, 10 years of grazing exclosure had a higher (*p* < 0.01) grass, herb and litter carbon stocks compared to 5 years exclosure and open grazing lands. The total carbon stock was higher for 10 years exclosure (75.65 t C ha^‐1^) than the 5 years exclosure (55.06 t C ha^‐1^) and in open grazing areas (51.98 t C ha^‐1^). Grazing lands closed for 10 years had a higher SOC, organic matter, total N, available P, and exchangeable K + and Na + compared to 5 year's exclosure and open grazing lands. Therefore, establishment of grazing exclosures had a positive effect in restoring degraded grazing lands, thus improving carbon sequestration potentials and soil nutrients.

## 1 INTRODUCTION

Grasslands cover 40% of the earth's land surface (Reynolds & Suttie, 2005; Wang & Fang, 2009). Grasslands are an essential ecosystem which plays a great role in the global carbon cycle and provides key ecosystem goods and services (Asner, Elmore, Olander, Martin, & Harris, 2004; FAO, 2010; Lal, 2004; Reynolds & Suttie, 2005; Wilcox & Thurow, 2006). However, grazing land resources are facing challenges like intense degradation as a consequence of deforestation, agricultural land expansion, and continuous heavy grazing (Lemenih, Karltun, & Olsson, 2005; Mengistu, Teketay, Hulten, & Yemshaw, 2005). In arid and semi‐arid grazing lands, overgrazing is one of the most important destructive factors, which causes to the increase of unpalatable species by destroying the most palatable species in the sward and reduce plant cover and biomass, thereby increase erosion hazard and reduce the overall productivity of the land (Bot & Benites, 2005; O'Connor, Haines, & Snyman, 2001).

The direct effect of livestock overgrazing includes consumption of the important plant species and soil trampling, which destroy the structure and composition of plant communities (Mekuria et al., 2015). Determination of herbivore density and proper distribution of livestock in grazing land are the most important issues in grazing land management (Liang et al., 2009) since vegetation biomass, vegetation height, and percentage of plant cover reduces with increasing grazing intensity (Abule, Smit, & Snyman, 2005; Tessema, Boer, Baars, & Prins, 2011; Tessema, Boer, & Prins, 2016). Light grazing increases aboveground biomass, canopy cover, and height of the species, but from a long‐term perspective, moderate grazing would balance the production of different species and livestock production (Wei, Hai‐Zhou, Zhi‐Nan, & Gao‐Lin, 2011). However, it may also lead to encroachment of unpalatable plants (Provenza, Villalba, Dziba, Atwood, & Banner, 2003).

Vegetation response to different grazing land management practices has been investigated in several studies (Bikila, Tessema, & Abule, 2016; Gebregerges, Tessema, & Birhane, 2017; Mekuria et al., 2015; Tessema et al., 2011; Yayneshet, Eik, & Moe, 2009) in which the results indicated that overgrazing of communal grazing lands causes a change in vegetation structure through decreasing the vegetation density and biomass. Continuous heavy grazing can also affect the carbon sequestration potentials of grazing lands through reduction of carbon accumulation in the soil systems (Alemu, 2012; Dlamini, Chivenge, & Chaplot, 2016; Mekuria, 2013; Solomon, Birhane, Tadesse, Treydte, & Meles, 2017). According to Mekuria et al. (2007) soils in areas excluded from gazing had a higher soil organic matter (SOM) contents compared to open grazed areas. Similarly, Conant, Cerri, Osborne, and Paustian (2017) in a new synthesis stated improved grazing management increases soil carbon. Thus, uncontrolled (open) grazing could result in severe degradation of both native vegetation and soil fertility in communal grazing lands in arid and semi‐arid environments (Yayneshet et al., 2009). However, a global review of Mcsherry and Ritchie (2013) showed that increasing grazing intensity increases soil organic carbon (SOC) in C4‐dominated and C4‐C3 mixed grassland, but decreased in C3‐dominated grasslands. Therefore, the effect of grazing intensity on SOC is highly context‐specific and depends on types of grasslands.

Restoration of degraded lands in arid and semi‐arid environments often involves excluding livestock from degraded sites (Mekuria, Veldkamp, Corre, & Haile, 2011; Mekuria et al., 2007; Mengistu et al., 2005; Yayneshet et al., 2009). According to Aerts, Nyssen, and Haile (2009) and Seyoum et al. (2015), exclosures are areas protected from human and domestic animal disturbances with the purpose of regenerating native vegetation and reducing land degradation of the formerly degraded communal grazing lands. Yayneshet et al. (2009) reported that exclosures can be effective in enhancing the composition, diversity, and density of vegetation on degraded grazing lands. Moreover, exclosures can be effective in restoring degraded soils and increasing soil carbon in the highlands of Tigray (Mekuria et al. (2011). Accordingly, rehabilitation of degraded communal grazing lands through establishing exclosures has become increasingly important in Tigray region, northern Ethiopia. Hence, approximately 1.5 million hectares of land have been excluded from grazing in the last three decades in the highlands of Tigray region (Seyoum et al., 2015). However, information on carbon sequestration and soil restoration potentials of degraded grazing lands after grazing exclusion in semi‐arid environments of Tigray region of Ethiopia is lacking. In the study area, the grazing exclosures were established in 2005 and 2010 in the lowlands of northern Ethiopia. Therefore, the objective of this study was (a) to assess the effect of grazing exclusion on biomass and soil carbon stocks and (b) to evaluate the impact of grazing exclusion on selected soil physicochemical properties.

## 2 MATERIALS AND METHODS

### 2.1 Study area

**Figure 1:**
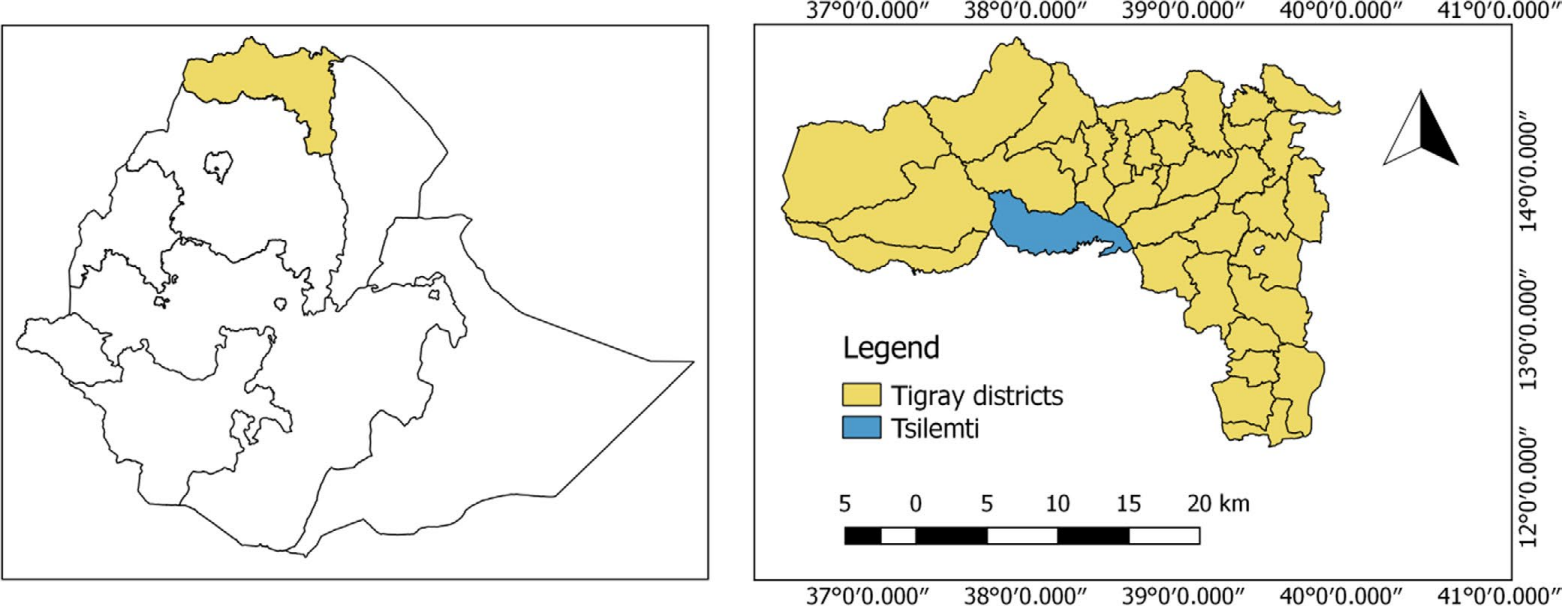
Map of the study area, Tselemti district, in Tigray region, northern Ethiopia

The study site was located in the semi‐arid areas of Tselemti district in the northwestern Tigray region of Ethiopia (Figure 1), which is located at 13°05′N latitude and 38°08′E longitude. The landscape of the district is characterized with flat plain plateau, mountainous valley, and some immediate break of slope with an altitude ranged between 800 and 2,870 m above sea levels. In Tselemti district, the most dominant soil groups are Cambisols, Fluvisols, Nitosols, and vertisols. Cambisols and Fluvisols constitute the largest soil group that covers about 75% of the soils in the district.

The mean maximum temperature varied between 33°C in April and 41.7°C in May, while the mean minimum temperature is between 15.8°C in December and 21.7°C in May. The dry season occurs from November to May, whereas the main rainy season occurs between June and September and follows a unimodal rainfall pattern with mean annual rainfall of 1,141.5 mm. The vegetation cover in the district includes Combretum–Terminalia and Acacia–Commiphora Woodlands which are characterized by small to moderate‐sized drought‐resistant trees and shrubs with fairly large deciduous leaves. *Anogeissus leiocarpus* (DC) Guill & Perri, *Dichrostachys cinerea* (L.) Wight & Am, *Dovyalis abyssinica* (A. Rich) Warb, *Oxythenanthera abyssinica* (A. Rich) Munro, *Boswellia papyrifera* (Del). Hochst, *Erytherina abyssinica* (DC) and *Balanites aegyptiaca* (L) Del are some of the dominant woody species in the study area (Gebregerges et al., 2017). The mean woody species density including seedlings encountered in open grazing land, 5‐year exclosure, and 10‐year exclosure were 391, 1,449, and 2,431 stems/ha, respectively (Gebregerges et al., 2017). In the rainy season, 16 different grass species were recorded and the area was dominated by grass species like *Pennisetum pedicellatum *Trin. (89.4 individuals/m^2^), *Hyparrhenia anthistirioides* (Hochst. ex A. Rich.) Andersson ex Stapf (78.3 individuals/m^2^), *Themeda triandra* Forssk. (68.8 individuals/m^2^), *Eragrostis cilianensis* (All.) Vignolo ex Janch. (51.1 individuals/m^2^), *Setaria pumila* (Poir.) Roem. & Schult. (47.6 individuals/m^2^) and *Digitaria velutina* (Forssk) P. Beauv. (24.4 individuals/m^2^) and other C4 plants like *Cynodon dactylon* (L.) Pers., *Cyperus rotundus *L., *Eleusine indica* (L.) Gaertn., *Panicum maximum *Jacq. and *Amaranthus spp* were the common herbaceous species observed in moderate number in the study area (Gebregerges et al., 2017).

### 2.2 Study site selection and field layout

**Figure 2:**
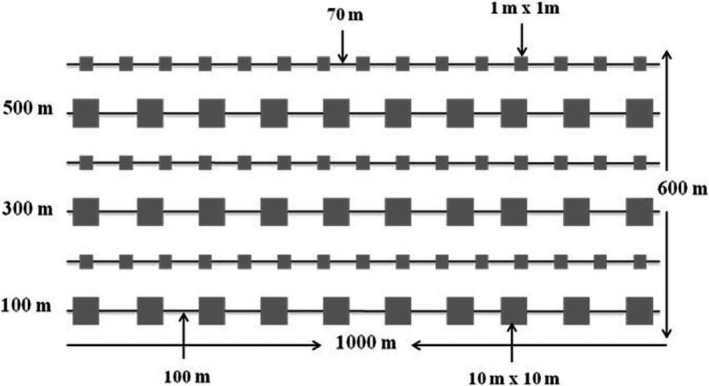
Sampling design and plot layout of the experimental site (Key: The small quadrats in the figure have 1 m × 1 m size and are allocated for herbaceous species as well as soil sampling. The larger squares in the figure have 10 m × 10 m size and are allocated for woody species sampling)

A field observation was made throughout the areas to be sampled prior to the field layout for vegetation and soil sampling. There were three grazing exclosures for each age class from which human and domestic animals interference was excluded. The grazing exclosures were well protected by guards who had been appointed by the community and were being paid by the government. Sampling sites within 5 years old exclosures, 10 years old exclosures, and open grazing lands were selected systematically for data collection. The size/area of the selected sites varied from 60 to 105 ha for open grazing lands and from 72 to 98 ha for grazing exclosures. The exclosures and open grazing lands were assumed to have been on similar conditions before the establishment of the exclosures. We selected three replicates for each exclosure age and open grazing land throughout the study area. The same site was divided into both exclosure and open grazing land due to the presence of high number of livestock and shortage of grazing land. Hence, the exclosure and the grazing lands were homogenous. We used systematic transect sampling technique to collect the vegetation and soil data within the three sampling sites (Figure 2) under each grazing land management. The first and the last transect lines were laid at least 100 m inside from the edge of the nearest adjacent grazing lands to avoid edge effects. The first sample plot was laid randomly and the others systematically at 100 m interval for larger plots (100 m^2^) and 70 m interval for smaller plots (1 m^2^) along the transect line. At each site, six parallel transect lines each with 1,000 m long at 200 m intervals from each other was established in each management practice. Plots measuring 10 m × 10 m were systematically taken for woody vegetation data collection according to previous studies (Dabasso, Taddese, & Hoag, 2014; Hasen‐Yusuf, Treydte, Abule, & Sauerborn, 2013; Mannetje & Jones, 2000). Small plots of 1 m^2^ were established for grass, herbs, and litter (GHL), as well as soil sampling in each transect line. A total of 270 sample quadrats were taken for sampling woody vegetation and 405 sample quadrats for measuring GHL and soil sampling during the study (Figure 2).

### 2.3 Sampling of woody vegetation and carbon stock determination

Data on woody vegetation were collected during September to October 2015. In each plot, every tree and shrub having a diameter of ≥2.5 cm at stump height (30 cm from the ground) and breast height (130 cm above the ground) circumference were measured with a meter tape and converted to diameter at breast height (DBH). The diameter was measured separately and considered as individual trees when the bole was branched at breast height or below. Moreover, in cases where tree/shrub boles buttressed, DBH was measured from the point just 5 cm above the buttresses. The diameters of multi‐stemmed shrubs were measured the same way as single‐stemmed trees according to Eshete and Ståhl (1998). The height of woody species was measured using calibrated bamboo stick having 7 m height graduated with 10 cm markings. Trees greater than 7 m in height were measured using clinometers.

The biomass and carbon stock of dominant tree/shrub was estimated using allometric equations developed for each tree/shrub species according to previous studies (Brown, 1997; Henry et al., 2011). The general allometric equation developed by WBISPP (2000) for all woody species were also used for estimating the aboveground woody biomass carbon stocks when species‐specific allometric equations were absent. Then, the aboveground woody biomass carbon is calculated from the aboveground biomass using a biomass carbon conversion factor of 0.5 (Liu et al., 2014). Moreover, the belowground biomass for trees and shrubs was estimated from root–shoot ratios by taking in to account the 27% of aboveground biomass of woody species (Penman et al., 2003).

### 2.4 Sampling of herbaceous vegetation and carbon stock determination

The aboveground biomass of herbaceous vegetation was measured in a 1 m^2^ quadrat from September to October 2015. Destructive sampling method was used for measuring the biomass of grasses and herbs by harvesting the whole fresh vegetation within each quadrat using hand shears. Clipped fresh samples together with litters were well‐mixed and weighed in the field using sensitive balance. Subsample of the total weight was separated and placed in a marked bag and taken to the laboratory to determine an oven‐dry‐to‐wet mass ratio that is used to convert the total wet mass to oven dry mass. The subsample was air dried and latter oven‐dried at Mekelle Soil Laboratory at 80°C for 24 hr according to Rau, Johnson, Blank, and Chambers (2009) until constant weight was obtained and finally re‐weighed for their dry weight using a sensitive balance with a precision of 0.1 g. Herbaceous vegetation carbon stocks were calculated as 50% of oven‐dried herbaceous biomass (Pearson, Walker, & Brown, 2005).

### 2.5 Sampling of soil parameters and laboratory analyses

Soil profile pits of 30 cm length and 50 cm width were opened in the center of the smaller (1 m^2^) plots. Soil samples were collected in each plot at three soil depths (0–10, 10–20, and 20–30 cm) from the four sides of the profile pits. Undisturbed soil samples were taken from each soil depth from the soil profile walls using a core sampler of 100 cm^3^ volume for soil bulk density (BD) determination. Equal volume of each sample from a given transect line were pooled and mixed together according to their depth, air dried and passed through a 2 mm sieve to separate debris and gravel. Finally, composite samples were divided into four equal parts, of which one was randomly chosen and stored in plastic bags, labeled, sealed, and transported to the soil laboratory for physical and chemical analyses. In the laboratory, soil samples were dried in an oven at 105°C for 24 hr for bulk density analysis. Bulk density was measured using the core method (Klute, 1986), and SOC was determined by Walkley–black method (Walkley & Black, 1934). Soil texture was analyzed by hydrometer method, pH using a pH‐meter in a 1:2.5 soils:water ratio. The percent soil organic matter (SOM) was calculated by multiplying the percent organic carbon by a factor of 1.724 (Brady & Weil, 1990). Total nitrogen was determined by the Kjeldahl method (Bremner & Mulvaney, 1982), available K and P were analyzed using ammonium acetate method and Olsen method (Olsen & Phosphorus, 1982), respectively. Mg and Ca were determined using atomic absorption spectrophotometer and flame photometer was used for K and Na (Jackson, 1958). EC was determined using the sodium saturation ratio (Reeuwijk, 1992), and cation exchange capacity (CEC) was determined using ammonium acetate method (Chapman & Norman, 1965).

### 2.6 Soil organic carbon stock assessment

Soil organic carbon was calculated using Pearson, Brown, and Birdsey (2007).

Soil organic carbon=bulk density×depth×%carbon

where,

$$\text{Bulk density}\left({\text{g cm}}^{-3}\right)=\frac{\text{oven dry mass}\left(\text{g}\right)}{\text{volume}\left({\text{cm}}^{3}\right)}$$

%Carbon = carbon concentration (%) determined in the laboratory following Walkley and Black (1934) method.

### 2.7 Estimation of total carbon stocks

The total carbon stock (C_t_) was calculated by summing the carbon stock values of the individual carbon pools of the land cover type using the following formula.

$$C_{t}\left(\text{ton/ha}\right)=\text{AGC}+\text{BGC}+\text{GHLC}+\text{SOC}$$

where,

AGC = aboveground carbon stock,
BGC = belowground carbon stock,
GHLC = grass, herb, and litter carbon stock and
SOC = soil organic carbon.

### 2.8 Statistical analysis

One‐way analysis of variance (ANOVA) using a general linear model (GLM) was applied to test for mean differences of biomass and carbon stock across grazing land management practices. Two‐way ANOVA was performed to test for mean differences of soil physicochemical properties across grazing land management and depth. Tukey HSD test was employed to investigate differences between means at *p* ≤ 0.05. Data were analyzed using SAS Software (SAS Inc., 2002).

## 3 RESULTS

### 3.1 Carbon stocks across grazing land management practices

**Figure 3:**
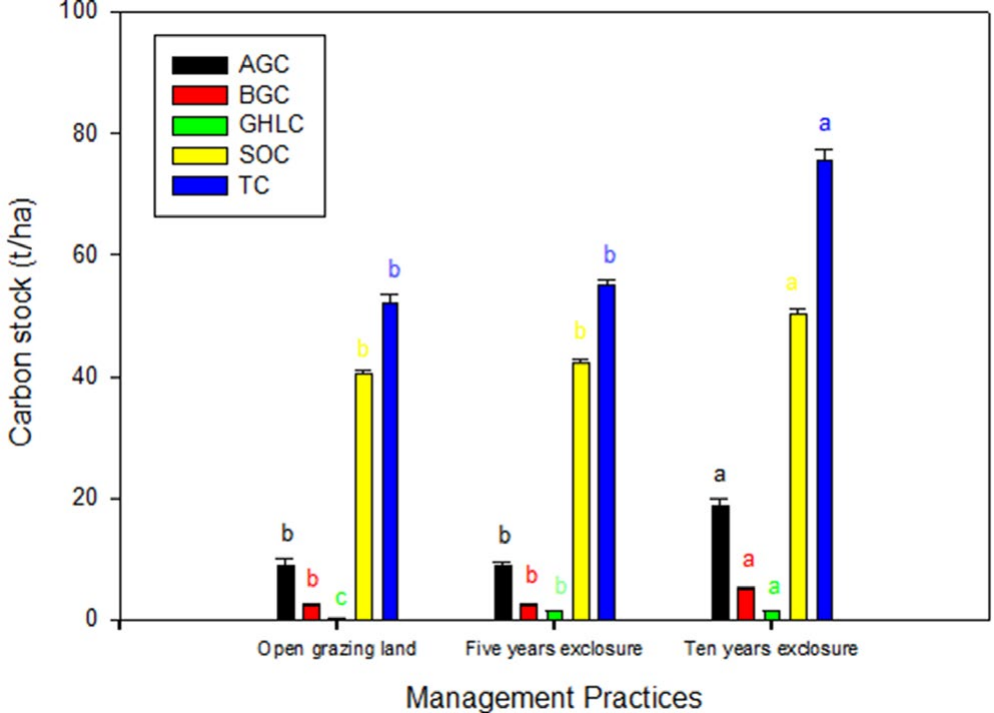
Carbon stock (ton/ha; mean ± *SE*) under the 5 and 10 years of grazing exclosures and open grazing lands

Significantly higher aboveground carbon stock was recorded in 10 years exclosure and lowest on open grazing land (Figure 3). The estimated mean belowground carbon stocks for the 10 years grazing exclosure was significantly higher (*p* < 0.001) than 5‐year grazing exclosure and open grazing lands (Figure 3). Grass, herb, and litter (GHL) carbon stocks significantly varied (*p* < 0.001) between open grazing lands, 5 and 10 years grazing exclosures, with the highest observed in the 10 years grazing exclosure, and lowest on open grazing land (Figure 3).

Soil organic carbon stocks showed a significant variation (*p* < 0.01) between the grazing land management practices, with the highest recorded in 10 years exclosure and lowest on open grazing land (Figure 3). However, there was no significant (*p* > 0.05) difference in SOC between the 5 years grazing exclosure and the open grazing lands.

The total carbon stock was significantly highest (*p* < 0.001) under the 10 years grazing exclosure as compared to 5 years grazing exclosure and the open grazing lands. Accordingly, the total carbon stocks for open grazing lands, 5 and 10 years of grazing exclosures were 52, 55, and 76 t C ha^‐1^, respectively (Figure 3). Higher total carbon stocks were stored in the soil than in biomass across all management practices.

### 3.2 Soil texture and bulk density

Clay and sand contents of the soil were not significantly (*p* > 0.05) affected by grazing land management, soil depth, and the interaction effects of grazing land management and soil depth. However, silt content of the soil was significantly (*p* < 0.05) affected by grazing land management (Table 1). Significantly highest silt content was recorded in 5 years of grazing exclosure as compared to open grazing land. Soil depth and the interaction effect of grazing land management and soil depth on silt content were not significant.

**Table 1:** Physical soil properties under the 5 and 10 years of grazing exclosures and open grazing lands

Parameters	Soil depth (cm)	Grazing land management practices		
		Open grazing land	Five years exclosure	Ten years exclosure
Clay	0–10	23.0 ± 4.16^a^	25.33 ± 1.85^a^	30.33 ± 6.56^a^
	10–20	27.67 ± 5.81^a^	25.67 ± 0.66^a^	30.33 ± 7.42^a^
	20–30	25.0 ± 4.16^a^	23.67 ± 1.76^a^	30.33 ± 5.8^a^
	Mean	25.22 ± 2.48^A^	24.89 ± 0.82^A^	30.33 ± 3.32^A^
Sand	0–10	50.0 ± 6.0^a^	38.70 ± 4.05^a^	42.7 ± 4.66^a^
	10–20	46.0 ± 7.02^a^	40.60 ± 1.33^a^	42.67 ± 4.66^a^
	20–30	51.3 ± 5.20^a^	44.70 ± 5.20^a^	38.70 ± 5.33^a^
	Mean	49.11 ± 3.16^A^	41.33 ± 2.13^A^	41.33 ± 2.54^A^
Silt	0–10	27.0 ± 2.30^a^	36.0 ± 3.0^a^	27.0 ± 4.61^a^
	10–20	26.33 ± 2.40^a^	33.67 ± 0.66^a^	27.0 ± 4.16^a^
	20–30	23.67 ± 1.33^a^	31.67 ± 3.52^a^	31.0 ± 2.31^a^
	Mean	25.67 ± 1.16^B^	33.78 ± 1.49^A^	28.33 ± 2.03^AB^
BD	0–10	1.31 ± 0.06^a^	1.16 ± 0.05^a^	1.03 ± 0.05^b^
	10–20	1.39 ± 0.04^a^	1.25 ± 0.04^a^	1.18 ± 0.08^ab^
	20–30	1.49 ± 0.04^a^	1.31 ± 0.04^a^	1.33 ± 0.05^a^
	Mean	1.40 ± 0.03^A^	1.24 ± 0.03^B^	1.18 ± 0.05^B^

Notes

Means with the same letters between rows (grazing land management) and column (depth) are not significantly different at *p* ≤ 0.05. Values are Mean ± *SEM* (standard errors of the mean).

Abbreviations: BD: bulk density (g/cm^3^); Clay: clay in %; Sand: sand in %; Silt: silt in %.

A reduction in grazing intensity reduced soil bulk density. Highest (*p* < 0.0001) bulk density was recorded in open grazing as compared to the 5 and 10 years exclosure (Table 1). Soil bulk density significantly (*p* < 0.05) varied across depths in the 10 years of exclosure, while no variation was recorded across depth in the open grazing land and 5 years exclosure. Highest soil bulk density was detected in the 20–30 cm depth in the 10 years exclosure.

### 3.3 Soil chemical properties

**Figure 4:**
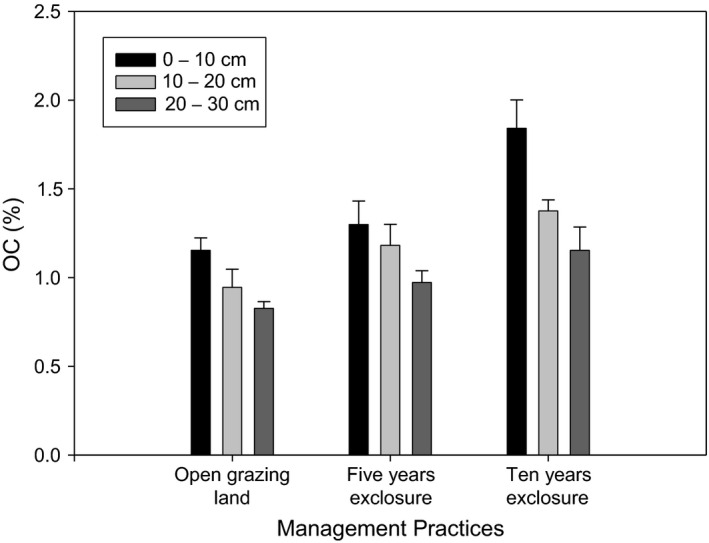
Organic carbon (%) under different grazing management practices

Soil organic carbon (OC) varied across grazing land management types, soil depth, and their interaction (*p* < 0.05) (Figure 4). Organic carbon ranged from 0.97 to 1.46, with the highest recorded in 10 years grazing exclosure, and the lowest on open grazing land. Significantly highest OC was observed in the 0–10 cm soil depth as compared to the 10–20 cm and 20–30 cm soil depths. However, there was no difference in OC in the 5 years grazing exclosure across depths.

**Figure 5:**
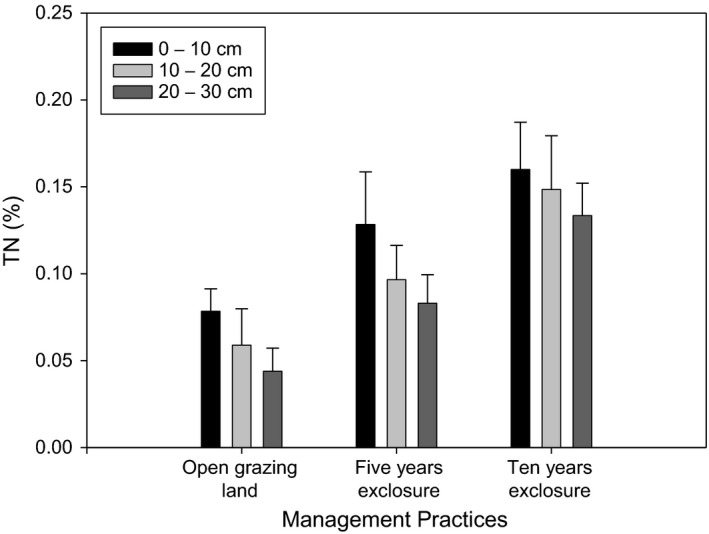
Total Nitrogen under the 5 and 10 years of grazing exclosures and open grazing lands

Total nitrogen (TN) significantly varied (*p* < 0.001) between the grazing land management practices, as low as to 0.06% in the open grazing lands to as high as 0.15% for the 10 years of grazing exclosure (Figure 5). Total nitrogen did not show variation across soil depth and the interaction effect of grazing management and soil depth. C/N ratio was significantly (*p* < 0.05) varied between grazing land management practices, with the highest observed in open grazing land and the lowest in 10 years exclosure.

**Figure 6:**
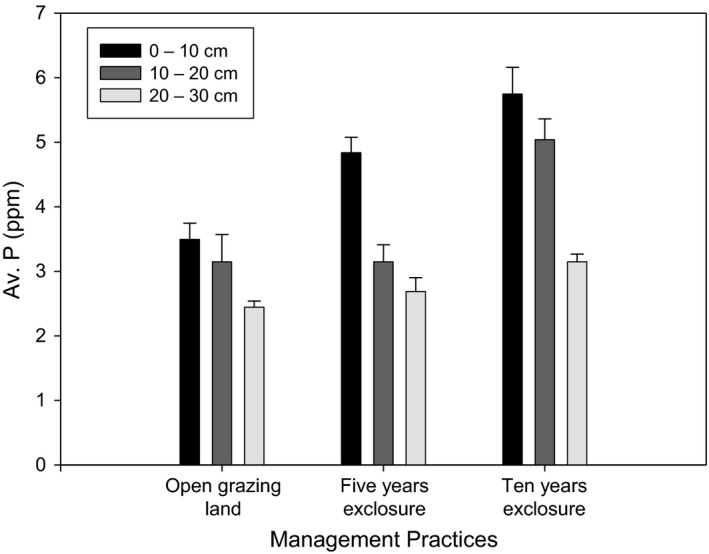
Available Phosphorus under the 5 and 10 years of grazing exclosures and open grazing lands

A reduction in grazing intensity did not show a difference in soil pH and electrical conductivity (EC). A reduction in grazing intensity improved available phosphorus (Av. P) concentration (*p* < 0.05). Significantly highest Av. P was recorded in 10 years grazing exclosure, while the lowest was observed in open grazing land (Figure 6). In the 5 and 10 years grazing exclosure, significantly highest Av. P was recorded in the topsoil (0–10 cm). Overall, significantly higher Av. P was observed in the 0–10 cm soil depth as compared to the 10–20 cm and 20–30 cm soil depths.

Mean soil Calcium (Ca) showed significant variation (*p* < 0.05) with grazing land management and the interaction of grazing land management and soil depth. Significantly (*p* < 0.05) highest soil Ca was recorded in the 10 years exclosure as compared to open grazing land. The lower soil depth (20–30) cm was recorded significantly higher carbon stock. In the 10 years grazing exclosure, significantly (*p* < 0.05) highest Ca was recorded in the 20–30 cm depth.

Soil magnesium (Mg), potassium (K), and sodium (Na) concentration showed significant variation between grazing land management, soil depth, and their interaction (Table 2). Significantly higher soil Mg was observed in the 10 years grazing exclosure as compared to the open grazing land. Soil Mg increased with increasing soil depths. In the open grazing land, significantly highest soil Mg was found in the 0–10 cm depth. Soil K significantly varied from 0.38 to 0.55 C mol (+)/kg, with the highest recorded in 10 years exclosure, and the lowest on open grazing land. From the soil depths, significantly higher K was recorded in the 0–10 cm and 10–20 cm as compared to the 20–30 cm soil depth. In the 5 years grazing exclosure, significantly higher K was recorded in the 0–10 cm and 10–20 cm as compared to the 20–30 cm soil depth. Significantly highest soil Na was observed in the 10 years grazing exclosure as compared to the open grazing land and 5 years grazing exclosure. Soil Na decreased with increasing soil depths. In the open grazing land, significantly higher soil Na was found in the 0–10 cm depth as compared to the 20–30 cm soil depth.

**Table 2:** Chemical soil properties under the 5 and 10 years of grazing exclosures and open grazing lands

Parameters	Soil depth (cm)	Grazing land management practices		
		Open grazing land	Five years exclosure	Ten years exclosure
C/N	0–10	15.57 ± 2.68^a^	11.79 ± 3.86^a^	12.09 ± 2.18^a^
	10–20	20.70 ± 6.69^a^	14.34 ± 5.18^a^	10.04 ± 1.92^a^
	20–30	22.70 ± 6.46^a^	13.39 ± 4.26^a^	9.07 ± 1.84^a^
	Mean	19.65 ± 2.99^A^	13.17 ± 2.26^AB^	10.40 ± 1.08^B^
PH	0–10	6.74 ± 0.24^a^	6.94 ± 0.07^a^	6.83 ± 0.06^a^
	10–20	6.94 ± 0.12^a^	6.93 ± 0.03^a^	6.87 ± 0.08^a^
	20–30	6.98 ± 0.15^a^	7.09 ± 0.03^a^	6.82 ± 0.08^a^
	Mean	6.89 ± 0.11^A^	6.98 ± 0.17^A^	6.84 ± 0.04^A^
EC	0–10	0.23 ± 0.04^a^	0.23 ± 0.01^a^	0.20 ± 0.02^a^
	10–20	0.25 ± 0.04^a^	0.26 ± 0.02^a^	0.23 ± 0.02^a^
	20–30	0.29 ± 0.05^a^	0.27 ± 0.02^a^	0.27 ± 0.01^a^
	Mean	0.26 ± 0.01^A^	0.26 ± 0.04^A^	0.24 ± 0.03^A^
Ca	0–10	11.00 ± 0.57^a^	11.67 ± 0.88^a^	12.67 ± 0.66^b^
	10–20	11.67 ± 0.66^a^	13.67 ± 0.66^a^	13.00 ± 0.57^b^
	20–30	13.00 ± 0.57^a^	14.00 ± 0.57^a^	15.67 ± 0.33^a^
	Mean	11.89 ± 1.23^B^	13.11 ± 0.22^AB^	13.78 ± 0.42^A^
Ex. Mg	0–10	3.33 ± 0.33^b^	4.00 ± 0.57^a^	5.00 ± 0.57^a^
	10–20	4.67 ± 0.33^a^	6.00 ± 0.57^a^	6.00 ± 0.57^a^
	20–30	5.00 ± 0.0^a^	5.67 ± 0.66^a^	6.67 ± 0.33^a^
	Mean	4.33 ± 0.33^B^	5.22 ± 0.55^AB^	5.89 ± 0.35^A^
Ex. K	0–10	0.45 ± 0.03^a^	0.53 ± 0.01^a^	0.60 ± 0.02^a^
	10–20	0.38 ± 0.03^a^	0.50 ± 0.00^a^	0.56 ± 0.04^a^
	20–30	0.32 ± 0.03^a^	0.42 ± 0.03^b^	0.49 ± 0.03^a^
	Mean	0.38 ± 0.04^C^	0.48 ± 0.031^B^	0.55 ± 0.024^A^
Na	0–10	0.39 ± 0.01^a^	0.43 ± 0.04^a^	0.49 ± 0.08^a^
	10–20	0.27 ± 0.05^ab^	0.26 ± 0.06^a^	0.42 ± 0.06^a^
	20–30	0.21 ± 0.01^b^	0.23 ± 0.05^a^	0.33 ± 0.05^a^
	Mean	0.29 ± 0.03^B^	0.31 ± 0.04^B^	0.41 ± 0.04^A^
CEC	0–10	35.40 ± 0.57^b^	39.93 ± 1.73^a^	39.40 ± 1.56^a^
	10–20	38.47 ± 0.74^a^	41.20 ± 1.52^a^	41.53 ± 0.96^a^
	20–30	40.46 ± 0.24^a^	42.73 ± 0.99^a^	43.67 ± 1.80^a^
	Mean	38.11 ± 2.8^B^	41.29 ± 0.83^A^	41.53 ± 0.96^A^

Notes

Means with the same letters between rows (grazing land management) and column (depth) are not significantly different at *p* ≤ 0.05. Values are Mean ± *SEM* (standard errors of the mean).

Abbreviations: Ca: calcium (Cmol (+)/kg); CEC: cation exchange capacity (meq/100 g of soil); C/N: carbon to nitrogen ratio; EC: electrical conductivity (mmhos/Cm); K: potassium (Cmol (+)/kg); Mg: magnesium (Cmol (+)/kg); Na: sodium (Cmol (+)/kg); pH: power of hydrogen ion (1:2.5).

Cation exchange capacity (CEC) showed significant (*p* < 0.05) variation between grazing land management, soil depth, and their interaction (Table 2). Significantly, higher CEC was observed in the 10 years exclosure and 5 years exclosure as compared to open grazing land. In the open grazing land, significantly higher CEC was recorded in the 10–20 and 20–30 cm as compared to 0–10 cm. Overall, soil CEC increased with increasing soil depths.

## 4 DISCUSSION

### 4.1 Carbon Stocks

A reduction in grazing intensity increased woody biomass carbon stocks. This was also observed in the study of Xiong, Shi, Zhang, and Zou (2016) and Yong Zhong, Zhao, and Zhang (2003) where significant biomass carbon was found as a result of grazing exclosure in grasslands of China. The highest woody biomass carbon stock in 10 years exclosures might be due to the availability of higher woody biomass in 10 years exclosure as compared with 5 years exclosure and open grazing lands. The lowest woody biomass carbon stocks in open grazing lands could be due to loss of carbon stocks as a result of overcutting and trimming of trees/shrubs by local people for fences, charcoal production, and other purposes. The mean biomass carbon stocks for the 10 years of grazing exclosure recorded in this study was similar to result of Alemu (2012). However, the woody biomass carbon stocks found in this study was two times lower compared to the woody biomass carbon stocks reported from rangelands exclosed for about 20 years in the southern parts of Ethiopia (Bikila et al., 2016). Moreover, the mean carbon stock in our study was four, five, three times higher than the carbon stocks reported in the Nile basin (Mekuria et al., 2015), in highlands of Northern Ethiopia (Mekuria, 2013), and in the shrublands of northern Kenya (Dabasso et al., 2014), respectively.

A higher grass, herbs, and litters (GHL) carbon stocks were found in the 10 years of grazing exclosure compared to the 5 years of grazing exclosures and open grazing lands in our study. This might be due to the fact that continuous heavy grazing in open grazing lands inhibits the growth of herbaceous layers and decrease aboveground herbaceous biomass through direct removal, leading to the depletion of GHL carbon stocks and soil nutrients. Our result is in agreement with previous studies (Bikila et al., 2016; Mekuria, 2013; Solomon et al., 2017; Xiong et al., 2016) who reported a higher herbaceous carbon stocks in exclosures as compared to open grazing lands. Moreover, the herbaceous carbon stocks reported in our study under the open grazing lands are almost similar to herbaceous carbon stocks in the untapped Boswellia woodlands in the northwestern lowlands of Ethiopia (Alemu, 2012). Our result indicated that exclusion of eroded and degraded grazing lands from animal interferences had a positive effect on the accumulation of herbaceous vegetation and litter carbon stocks.

The 10 years of grazing exclosures had the highest SOC, whereas the lowest SOC was recorded in the open grazing lands in our study. The differences in SOC stocks between the 10 years of grazing exclosure and open grazing lands could be as a result of the increased vegetation biomass and the subsequent production and decomposition of litterfall from this vegetation that would add organic matter into the soil systems. Thus, grazing lands with more aboveground vegetation biomass contribute more to the soil carbon sequestration potential as compared to grazing lands having less aboveground vegetation biomass. The findings of the present study are in agreement with the findings of Bikila et al. (2016) and Mekuria (2013) in which they found higher SOC for exclosures than the open grazing areas. Similarly, Li, Zhao, Chen, Luo, and Wang (2012) reported grazing exclusion is a positive way to restore desertified ecosystems and has a high potential for sequestering soil carbon in the semi‐arid Horqin Sandy Land. A meta‐analysis by Dlamini et al. (2016) reported that grassland degradation significantly reduced SOC stocks by 16% in dry climates (<600 mm) compared to 8% in wet climates (>1,000 mm). Improved grazing management, fertilization, sowing legumes and improved grass species, irrigation, and conversion from cultivation all contributed to grassland improvement. A new synthesis by Conant et al. (2017) also stated that improved grazing management in combination with other factors tends to lead to increased soil C, at rates ranging from 0.105 to more than 1 Mg C ha^‐1^ year^‐1^. In contrary to our result, soil organic carbon showed no significant differences between grazed and nongrazed conditions in NW Patagonia, Argentina (Nosetto, Jobbágy, & Paruelo, 2006) and in southern Ethiopian rangelands (Aynekulu et al., 2017). A global review by Mcsherry and Ritchie (Mcsherry & Ritchie, 2013) showed that increasing grazing intensity increased SOC by 6%–7% on C4‐dominated and C4–C3 mixed grasslands, but decreased SOC by an average of 18% in C3‐dominated grasslands. Carbon stocks in the soil layers 0–5 and 5–15 cm under grazed grassland were significantly larger than in the ungrazed grassland Tibetan montane pasture (Hafner et al., 2011). Shrestha and Stahl (2008) found no variation in soil organic carbon due to grazing exclusion at three of their four study sites, where exclosures had been established more than four decades earlier, in the semi‐arid sagebrush steppe of Wyoming. The discrepancies among these studies likely resulted from differences in climate among study sites and in specific soil characteristics. The degree of degradation caused by grazing before the exclosure was implemented, the manure inputs from livestock, the balance between livestock stocking rate and carrying capacity, and the original and postgrazing vegetation communities (Li et al., 2012; Reeder & Schuman, 2002) could contribute to the variations.

The total carbon stocks were highest in the 10 years of grazing exclosure compared to the 5 years of grazing exclosure and open grazing lands in this study. This was confirmed by the study of Mekuria (2013) stated ecosystem carbon stock increased with increase in exclosure age in communal grazing lands of Ethiopia. Under exclosure conditions, vegetation restoration and litter accumulation significantly increased total carbon storage, and thus sequestration of atmospheric carbon (Yong Zhong et al., 2003). However, (Nosetto et al., 2006) found that grazing exclosures did not result in significant changes in the total carbon storage in comparison with the adjacent grazed stands, suggesting a slow ecosystem recovery in the time frame of their study (15 years of exclusion) in NW Patagonia, Argentina.

Moreover, across the grazing land management practices, a higher total carbon stock was stored in soil than in the aboveground vegetation. According to Girmay, Singh, Mitiku, Borresen, and Lal (2008), more than 90% of the total carbon stocks were contributed from SOC in wooded grassland of northern Ethiopia.

### 4.2 Soil physicochemical properties between land management practices and soil depths

The results of the soil texture showed significantly highest silt content in 5 years exclosure as compared to the open grazing land and 10 years exclosure. Despite the differences, the results did not show any relationship between grazing management and soil texture. Soil texture is one of the inherent soil characteristics that changes rarely (Khademolhosseini & Jahromi, 2014). Therefore, the difference in silt content among the grazing management might be due to other factors instead of grazing management. However, the highest sand percentage observed in the open grazing lands might be due to the decrease in ground cover as a result of continuous heavy grazing, which accelerates erosion of fine soil particles (Pei, Fu, & Wan, 2008; Yong‐Zhong, Yu‐Lin, Jian‐Yuan, & Wen‐Zhi, 2005).

Areas excluded from grazing had a lower soil bulk density than open grazing lands while there is no significant variation between young and old exclosures in our study, indicating that excluding of livestock from degraded grazing areas significantly decreased soil bulk density. In line with this study, Pei et al. (2008) and Liu, Wu, Su, Gao, and Wu (2017) also found that soil bulk density decreased after 6 years of exclosure and 10 years of exclosure in degraded Alxa desert steppe and Xilin Gol grassland of Inner Mongolia, China, respectively. Li et al. (2012) also showed that along the age sequence of grazing exclosure for 8, 13, and 26 years, bulk density in the top 20 cm of the soil decreased in Inner Mongolia, northern China. Similar results were observed in Steffens, Kölbl, and Totsche (2008) indicated bulk density significantly increased with increase in grazing intensity in a semi‐arid steppe of Inner Mongolia. In contrary, Aynekulu et al. (2017) stated that excluding of grazing land had no effect on bulk density in Southern Ethiopian rangelands. The decrease in bulk density in grazing exclosures may increase soil aeration, water absorption, and water holding capacity and reduces runoff (Kozlowski, 1999; Lal & Kimble, 2001).

Significantly highest OC, TN%, and AP were recorded in the 10 years of grazing exclosure. In line with this study, Pei et al. (2008) found OC, TN, and AP increased significantly with exclosure period. Li et al. (2012) and Mekuria (2013) also stated grazing exclosure had a potential to restore soil nutrients. A meta‐analysis by Xiong et al. (2016) found that soil available nitrogen and soil available phosphorus increased by 52.0% and 21.7%, respectively, in grasslands of China. The highest OC, TN%, and AP recorded from the 10 years of grazing exclusion could be due to the higher accumulation and decomposition of litters into the soil. The results for the OC, TN% and AP was in agreement with the reports of Yimer, Alemu, and Abdelkadir (2015) who stated that the relative increase in the soil parameters in exclosures is due to the management establishment and subsequent increased organic matter accumulation derived from litterfall from the trees/shrubs and herbaceous species biomass and from reduced soil erosion through effective ground cover. Besides, the increases in canopy cover with the increase in exclosure duration could decrease soil nutrient losses by reducing the erosive impact of raindrops and soil erosion (Girmay, Singh, Nyssen, & Borrosen, 2009; Mekuria et al., 2009). In contrary to our result, Aynekulu et al. (2017) found similar value of OC concentration and total nitrogen between exclosure and the adjacent open area in southern Ethiopia rangelands. Yong‐Zhong et al. (2005) observed an insignificant increase in soil organic OC and TN to a depth of 15 cm after 5 years of exclosure in degraded grassland in the Horqin Sandy Land.

Mean soil Calcium (Ca) showed significant variation (*p* < 0.05) with grazing land management and the interaction of grazing land management and soil depth. Significantly (*p* < 0.05) highest soil Ca was recorded in the 10 years exclosure as compared to open grazing land. The lower soil depth (20–30) cm was recorded significantly higher carbon stock. In the 10 years grazing exclosure, significantly (*p* < 0.05) highest Ca was recorded in the 20–30 cm depth.

Soil magnesium (Mg), potassium (K), and sodium (Na) concentration showed significant variation between grazing land management, soil depth, and their interaction. Significantly higher soil Mg, K, and Na was observed in the 10 years grazing exclosure as compared to the open grazing land. Similarly, Li et al. (2012) found that along the age sequence of grazing exclosure for 8, 13, and 26 years, K increased in the top 20 cm of the soil decreased in Inner Mongolia, northern China.

Cation exchange capacity (CEC) showed significant (*p* < 0.05) variation between grazing land management, soil depth, and their interaction. Significantly higher CEC was observed in the 10 years exclosure and 5 years exclosure as compared to open grazing land. In the open grazing land significantly highest CEC was recorded in the 10–20 and 20–30 cm as compared to 0–10 cm. Overall, soil CEC increased with increasing soil depths. In line with this study, Mekuria and Aynekulu (2013) and Mekuria (2013) found that exclosures showed significantly higher CEC than the adjacent grazing lands in northern Ethiopia.

## 5 CONCLUSIONS

The establishment of area exclosures on degraded communal grazing lands had positive effect in restoring vegetation biomass, carbon sequestration potentials, and soil nutrients of eroded communal grazing lands. The aboveground biomass and carbon stocks increased with duration of grazing exclusion; however, the open grazing lands and 5 years of grazing exclosure did not differ significantly in our study. A similar pattern was observed for belowground carbon stocks and soil organic carbon stocks, that is, the grazing lands excluded for 10 years from grazing differed significantly with both the 5 years closed area and the open grazing lands. The grass, herbs, and litter carbon stocks were the highest in the 10 years of grazing exclosure, amounting almost more than five times the value recorded in the open grazing lands. Similarly, the overall total carbon stock was highest for the 10 years of grazing exclosure followed by the 5 years of grazing exclosure and open grazing areas. In the present study, higher total carbon stock was stored in soil than in the aboveground vegetation across all grazing land management practices. Therefore, establishment of area exclosures needs to be widely practised in the semi‐arid areas of the region to enhance vegetation biomass, carbon sequestration potentials, and soil nutrient contents. Moreover, further studies on temporal and spatial vegetation biomass and carbon stocks need to be thoroughly investigated to capture the whole dynamics of the grazing land ecosystems under various regimes of grazing exclusions in arid and semi‐arid environments.

## CONFLICTS OF INTEREST

The authors declare no conflict of interest.

## AUTHOR CONTRIBUTIONS

T.G. conceived and designed the study; T.G. collected the data. T.G, E.B, Z.K.T, and N.S analyzed the data and wrote the paper; E.B., Z.K.T, and N.S. critically reviewed the paper and provided comments on the contents and structure of the paper.

## DATA AVAILABILITY

Excel files containing all carbon stock and soil properties data have been submitted to Dryad https://doi.org/10.5061/dryad.v7t77ts.
